# Natural language processing analysis of the theories of people with multiple sclerosis about causes of their disease

**DOI:** 10.1038/s43856-024-00546-3

**Published:** 2024-06-24

**Authors:** Christina Haag, Nina Steinemann, Vladeta Ajdacic-Gross, Jonas Tom Thaddäus Schlomberg, Benjamin Victor Ineichen, Mina Stanikić, Holger Dressel, Paola Daniore, Patrick Roth, Sabin Ammann, Pasquale Calabrese, Christian Philipp Kamm, Jürg Kesselring, Jens Kuhle, Chiara Zecca, Milo Alan Puhan, Viktor von Wyl

**Affiliations:** 1 Epidemiology, Biostatistics and Prevention Institute, University of Zurich, Zurich, Switzerland; 2 Institute for Implementation Science in Health Care, University of Zurich, Zurich, Switzerland; 3 Psychedelic Research & Therapy Development, Department of Adult Psychiatry and Psychotherapy, Psychiatric University Clinic Zurich, University of Zurich, Zurich, Switzerland; 4 Center for Reproducible Science, University of Zurich, Zurich, Switzerland; 5 Clinical Neuroscience Center, University of Zurich, Zurich, Switzerland; 6 Division of Occupational and Environmental Medicine, University of Zurich and University Hospital Zurich, Zurich, Switzerland; 7 Digital Society Initiative, University of Zurich, Zurich, Switzerland; 8 Department of Neurology & Brain Tumor Center, University Hospital and University of Zurich, Zurich, Switzerland; 9 Division of Molecular and Cognitive Neuroscience, Neuropsychology and Behavioral Neurology Unit, University of Basel, Basel, Switzerland; 10 Neurocentre, Lucerne Cantonal Hospital, Lucerne, Switzerland; 11 Department of Neurology, Inselspital, Bern University Hospital, University of Bern, Bern, Switzerland; 12 Department of Neurology and Neurorehabilitation, Rehabilitation Center Valens, Valens, Switzerland; 13 Department of Neurology, University Hospital Basel and University of Basel, Basel, Switzerland; 14 Multiple Sclerosis Center and Research Center for Clinical Neuroimmunology and Neuroscience Basel, Departments of Biomedicine and Clinical Research, University Hospital Basel and University of Basel, Basel, Switzerland; 15 Multiple Sclerosis Center, Neurocenter of Southern Switzerland, EOC, Lugano, Switzerland; 16 Faculty of Biomedical Sciences, Università della Svizzera Italiana, Lugano, Switzerland

**Keywords:** Multiple sclerosis, Multiple sclerosis

## Abstract

**Background:**

While potential risk factors for multiple sclerosis (MS) have been extensively researched, it remains unclear how persons with MS theorize about their MS. Such theories may affect mental health and treatment adherence. Using natural language processing techniques, we investigated large-scale text data about theories that persons with MS have about the causes of their disease. We examined the topics into which their theories could be grouped and the prevalence of each theory topic.

**Methods:**

A total of 486 participants of the Swiss MS Registry longitudinal citizen science project provided text data on their theories about the etiology of MS. We used the transformer-based BERTopic Python library for topic modeling to identify underlying topics. We then conducted an in-depth characterization of the topics and assessed their prevalence.

**Results:**

The topic modeling analysis identifies 19 distinct topics that participants theorize as causal for their MS. The topics most frequently cited are Mental Distress (31.5%), Stress (Exhaustion, Work) (29.8%), Heredity/Familial Aggregation (27.4%), and Diet, Obesity (16.0%). The 19 theory topics can be grouped into four high-level categories: physical health (mentioned by 56.2% of all participants), mental health (mentioned by 53.7%), risk factors established in the scientific literature (genetics, Epstein-Barr virus, smoking, vitamin D deficiency/low sunlight exposure; mentioned by 47.7%), and fate/coincidence (mentioned by 3.1%). Our study highlights the importance of mental health issues for theories participants have about the causes of their MS.

**Conclusions:**

Our findings emphasize the importance of communication between healthcare professionals and persons with MS about the pathogenesis of MS, the scientific evidence base and mental health.

## Plain Language Summary

Multiple sclerosis (MS) is a disease that affects the brain and spinal cord, causing a wide range of symptoms. Our study investigated what people living with the disease think causes MS. We analyzed the replies given by 486 people who were questioned about their MS to look for patterns in the responses. We identified 19 distinct themes, notably mental and work-related stress, genetics, and dietary factors, which we grouped into 4 categories: physical health, mental health, established scientific risk factors, and chance. We found that mental health problems were viewed as a key factor for MS. Our work highlights the need for healthcare professionals to have transparent conversations with people with MS about what is known about the disease course and potential causes. In addition, it highlights the importance of fully informing and supporting people with MS regarding their mental health.

## Abstract (web summary)

Haag et al. examine the theories of persons with multiple sclerosis (MS) about the causes of MS using natural language processing techniques. Their study identifies unique topics and highlights the role of mental health and the need for an open dialogue about the scientific and mental health aspects of MS.

## Introduction

Multiple sclerosis (MS) is a progressive neurodegenerative disease that is characterized by a wide range of physical and mental symptoms^1^. Typical symptoms include gait difficulty, mental and physical fatigue, vision problems, vestibular disorders, incontinence, numbness or tingling in different parts of the body. While the causes of MS remain unclear, genetic and environmental risk factors have been consistently associated with MS, as evidenced by several rigorous meta-analyses. The genetic theory is mainly based on the observed familial aggregation of MS, with the most commonly associated genetic factor being the HLA-DR1*15:01 allele^2^. Established environmental factors include tobacco smoking, low vitamin D levels, low exposure to ultraviolet radiation, and infection with Epstein-Barr virus (EBV), a human lymphotropic herpes virus^3–8^.

A key question is how persons with MS understand the causes of their disease and how they deal with the question of why they have MS. These questions are key since evidence-based treatments may lose legitimacy in the eyes of the patient if a given treatment does not align with their individual theory. In this context, the Health Belief Model is a central framework that theorizes health behavior as a function of beliefs about disease severity, presumed vulnerability, benefits associated with a health behavior, and perceived barriers to engaging in the given health behavior^9^. Consistently, previous research has found that the self-efficacy and scientific accuracy with which persons with MS understand their disease predict their wellbeing – for example, through increased effectiveness of their coping strategies^10,11^. The Cognitive Theory of Adaptation assumes that serious somatic illness poses three major challenges for individuals: maintaining self-esteem, finding meaning and significance in the illness, and achieving a subjective sense of control over the course of the illness^12^. Understanding the disease as a consequence of a particular lifestyle or stressful life experience may help persons with MS to regain a sense of control over their lives, despite the unpredictability that comes with MS^13^.

While theories of persons with MS often share common ground with accepted scientific theories, they can also incorporate additional explanatory components that have not yet been thoroughly examined or explored. A qualitative, large-scale study of such individual theories of MS would therefore provide valuable insight into this otherwise unexplored topic. In addition, a comprehensive, qualitative study of such common theories would be of great value in optimizing support for persons with MS, both in terms of addressing unmet information needs and promoting individuals’ coping skills. The practical value of large-scale text exploration using the well-established topic modeling approach has been well documented in previous research^14^, including studies related to MS. For instance, the My Life with MS study examined the life stories of over 1000 participants and identified eight key topics, covering established clinical aspects as well as less explored areas such as work and relationships^15^. A study using natural language processing to explore the daily-life impact of the first lockdown on people with MS found extensive and varied experiences^16^. However, manual content analysis is still widely used. For example, a 2023 study using free-text data examined the impact of the COVID-19 pandemic on persons with MS in Australia^17^. Recent approaches to topic modeling are based on large-scale language models^18^. These recent developments have been driven by the emergence of transformer(-based) language models, which have dramatically accelerated the performance and computational speed of previously complex natural language processing tasks^19^, thus facilitating the identification of topics in free-text data with high quality and ease^20^.

Here we present the results of a survey on theories that persons with MS have about the causes of their disease, which was part of a larger questionnaire focusing on risk factors associated with MS. The survey was designed and conducted by the Swiss MS Registry (SMSR) in close collaboration with and at the initiative of persons with MS. We examine the broad categories into which individual theories could be grouped as well as the prevalence of each theory. We expected a variety of theories for which participants would draw on their life experiences, and that many of them would also mention the well-established risk factors for developing MS (genetics, EBV, smoking, vitamin D deficiency). Topic modeling analysis identifies 19 distinct topics that participants theorize as causal for their MS which can be grouped into four high-level categories: physical health, mental health, risk factors established in the scientific literature and fate/coincidence.

## Methods

### Study design and participants

Participants were recruited from the SMSR, an ongoing longitudinal patient-centered study in Switzerland funded by the Swiss MS Society^21^. The study was approved by the Ethics Committee of the Canton of Zurich (PB-2016-00894, BASEC-no: 2019-01027). All SMSR participants provided written informed consent prior to their participation. The SMSR’s overarching aim is to give persons with MS a voice and to actively involve them in research projects (referred to as citizen science), as they are experts in their particular disease symptomatology. The registry combines the accumulated knowledge and experience into a single database with great potential for in-depth research into MS and the further development of treatment and care. A unique feature of the SMSR is that persons with MS not only contribute to the registry database as participants – they are also part of the decision-making bodies of the registry and are involved in the selection of research questions, study design, and communication of research results. Their knowledge and experience are thus fully integrated into the SMSR’s activities.

The present research is based on a survey assessing participants’ individual theories about the causes of their MS. The survey was part of the SMSR’s Risk Factors Project which was launched in 2020 and data collection continued until March 2023. The overall aim of the project was to comprehensively assess risk factors for the onset and progression of MS. The risk factor assessment began with a survey of participants’ individual theories about the causes of their MS, which can be found in Supplementary Note 1. The present study is based on the following two survey questions. Question 1: Have you made any assumptions about how you developed MS? What do you think? Question 2: Are there any specific risk factors that come to your mind? If so, why? Question 1 was designed as an introduction to familiarize participants with the topic of theories about the cause of their MS, while question 2 was designed as a follow-up question to elicit more detail about the specific nature of the risk factors and possible underlying mechanisms. However, many participants provided a comprehensive answer to question 1 that also covered question 2. In this case, participants either left question 2 blank or briefly repeated what they had written before. Given these response patterns, the text data from both questions were combined for subsequent analysis.

### Statistics and reproducibility

Below we provide all the information about how we proceeded with the natural language processing analysis. For reproducibility purposes, we provide additional information on the analysis in Supplementary Method 1 and the analysis code in Supplementary Method 2.

### Text data preprocessing

The SMSR conducts its assessments in the three official Swiss languages German, French, and Italian. All text entries underwent manual spell-checking conducted by native-level speakers of each respective language. Since most of the text data was in German, French and Italian text data were automatically translated into German. All analyses were performed in German. Text data presented in graphs were translated into English for this purpose. For automatic text translation, we used the Hugging Face transformers library in Python^22^ and open-source language models for translation (https://huggingface.co/Helsinki-NLP). All translations were reviewed by native speakers in French/German, and Italian/German. A detailed description of text preprocessing is provided in Supplementary Method 1.

### Topic modeling with BERTopic

**Fig. 1:**
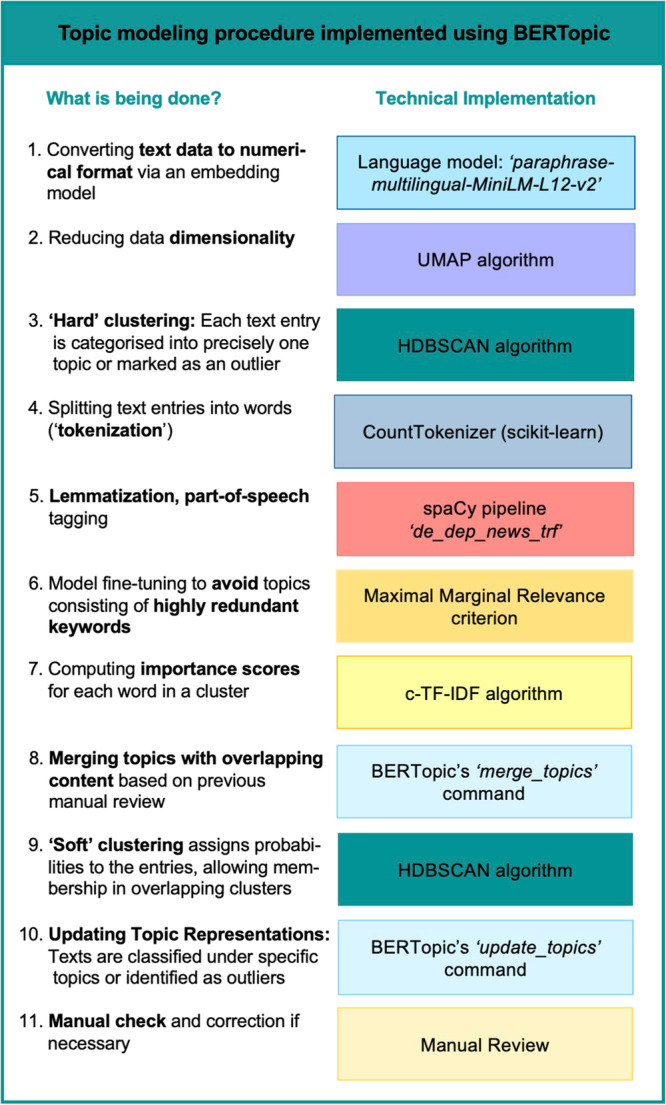
Modeling procedure implemented using BERTopic. This figure outlines the steps and technical implementations in the BERTopic model for topic modeling. The process includes converting textual data to numerical format, dimensionality reduction, clustering, tokenization, lemmatization, fine-tuning, calculating importance scores, merging topics, updating topic representations, and manual review. Abbreviations. c-TF-IDF: class-based Term Frequency – Inverse Document Frequency; HDBSCAN: Hierarchical Density-Based Spatial Clustering of Applications with Noise; spaCy: open-source Python library for Natural Language Processing; UMAP: Uniform Manifold Approximation and Projection for Dimension Reduction.

The individual steps of the topic modeling procedure using BERTopic are illustrated in Fig. 1. To identify common types of life events, we used the state-of-the-art Python library BERTopic^18^, which leverages transformer-based language models in several steps of the modeling process. All steps were performed in Python, version 3.7, using the PyCharm environment, version 2021.3.2.

The main analysis concerns the training of the topic model. We first transformed the text data into numerical representations by using the well-established sentence transformer model paraphrase-multilingual-MiniLM-L12-v2^23^, which is suitable for German language. We then reduced the dimensionality of the data using the built-in UMAP algorithm^24^ and employed BERTopic’s built-in HDBSCAN clustering algorithm^25^ for subsequent clustering. The latter step can be considered a hard clustering approach, as each text entry is assigned to precisely one topic or marked as an outlier. We then tokenized the data using the well-established CountVectorizer from the Scikit-Learn library^26^. When tokenizing, we lemmatized all words, meaning that we converted them to a meaningful root word (e.g., treatments to treatment). Since nouns were the most informative for our research aim, we next extracted all lemmatized nouns from the text segments using the part-of-speech tagging functionality of the spaCy pipeline de_dep_news_trf^27^. To assign an importance score to each unique word within the topic representations, BERTopic implements the statistical metric class-based term frequency-inverse document frequency. Finally, we fine-tuned the model using the Maximal Marginal Relevance^28^ criterion to avoid topics consisting of excessively redundant keywords (such as smoker, smoke, smoking). A few topics were then merged due to substantial overlap in content. Using the Python WordCloud library^29^, we then visualized the final topic representations in a separate graph for each topic, where the size of a word corresponds to its importance score.

Fitting the HDBSCAN cluster model in BERTopic does not force all text data into clusters, and a number of text entries are initially assigned to an outlier category. This procedure ensures high topic interpretability as the topic representation is based on text data with a high probability of belonging to a particular cluster, while text data with low probability values are initially considered as outliers (referred to as hard clustering). We then used a soft clustering approach to assign initially unclassified text data to the most appropriate cluster, assigning each text entry to the topic category for which it had the highest probability^25^. We used a threshold of *p* < 0.05, and text data that had probability values below 0.05 to belong to any cluster were still considered outliers. All classifications and outliers were then manually checked by CH and corrected where necessary. Of the 1494 text segments, the category assignment was corrected in 183 cases corresponding to an accuracy of 87.8%, by applying evaluation criteria for classifiers^30^, where accuracy is defined as the number of correct classifications divided by the total number of classifications. A further 42 text segments had a second, and in a few cases a third category, which was also added manually.

### Topic co-occurrence

We determined the occurrence of topics (i.e., their presence or absence) per individual using Pearson correlations.

### Thematic analysis for validation

Given the unsupervised nature of our topic modeling analysis, we conducted a thematic analysis on the entire text dataset as a benchmark for validation. The analysis was performed by JTTS with input from VvW.

### Reporting summary

Further information on research design is available in the Nature Portfolio Reporting Summary linked to this article.

## Results

### Sample characterization

All SMSR participants (n = ~2700, status March 2023) were invited to participate in the present study. A total of 603 individuals participated in the survey about theories of the origin of their MS. Of these, 107 participants either provided no response or indicated in their free-text response that they did not have a hypothesis about the cause of their illness and were therefore not included in this analysis. Participants who reported having no theories often indicated that they would not benefit from thinking about such issues, preferring instead to focus on the present. The responses of a further 10 participants could not be classified, either because they indicated that they had a theory but provided no further details, or because their responses did not directly address the questions (but included, for example, symptom descriptions of the early stages of their MS).

This resulted in a final sample of 486 participants whose text responses form the basis of this study. The mean age of the final study sample participants was 52.15 years (standard deviation, SD = 12.48 years; range: 21–86) and 80.3% were female. On average, participants were diagnosed 12.96 years ago (SD = 9.50). Most participants had relapsing-remitting MS (*n* = 339). The remaining participants were diagnosed with secondary progressive MS (*n* = 59), primary progressive MS (*n* = 47), clinically isolated syndrome (*n* = 14), or the type of MS was unknown at the time of the survey (*n* = 27). Sociodemographic and clinical characteristics of the 486 individuals included in the analysis and those not included (*n* = 938) due to either lack of MS theory (*n* = 107) or non-response to the survey (*n* = 831), were assessed at the time of their enrollment in the registry. Individuals included in this analysis were more often female, *p* = .001, and slightly older (mean=48 years) than those not included (mean=45 years, *p* = .001). A comprehensive characterization of responders and non-responders is provided in Supplementary Data 3.

Of the 486 participants, 78.6% provided their text entries in German (*n* = 382), 17.7% in French (*n* = 86), 3.3% in Italian (*n* = 16), and 0.4% in English (*n* = 2). The average length of the text entries was 27.46 words (SD = 25.46) per participant after cleaning as described in the Methods section.

### Theories about the causes of MS – topic modeling analysis

The topic modeling analysis resulted in 19 distinct topics and a category consisting or micro-topics and unspecific mentions. The number of topics per individual ranged from 0 to 13, with an average of 2.27 topics (standard deviation: 1.54). Seven individuals did not mention any of the main topics, with their theories falling into the micro-topic category or as unspecific or rare mentions. A quarter of the participants mentioned a maximum of one topic (1st quartile), half of them discussed no more than two topics (median), and three quarters mentioned up to three topics (3rd quartile).

**Fig. 2:**
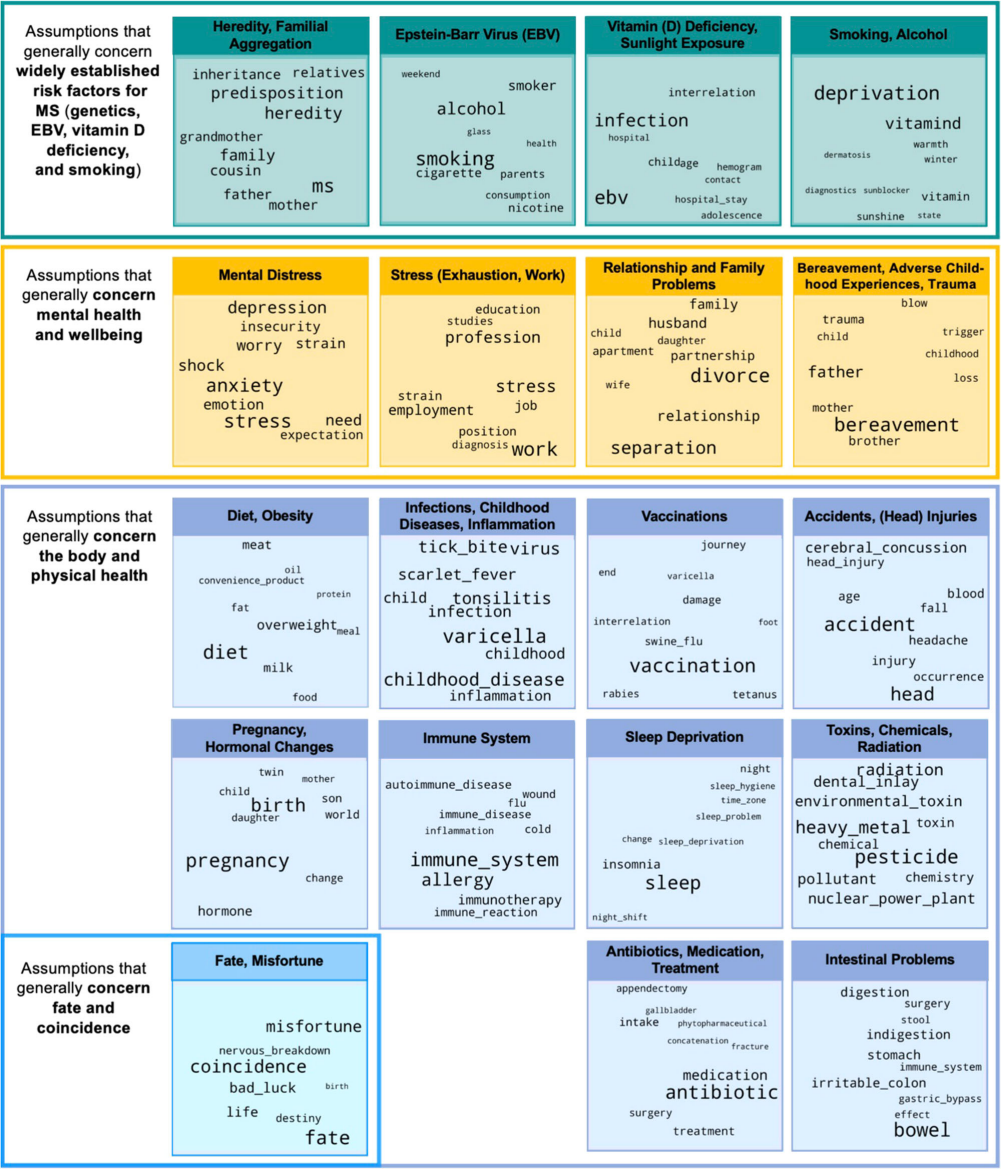
Persons with MS’ theories about the causes of their disease - results of the topic modeling analysis. The figure shows the 19 distinct topics identified by the topic modeling analysis using BERTopic. The 19 topics have been grouped into four high-level categories: physical health (mid-blue), mental health (orange), risk factors identified in the scientific literature (turquoise), and fate/coincidence (light blue). For each of the 19 topics, the key words that define a given topic are presented in the form of a word cloud. In each word cloud, the size of the word corresponds to the relative importance of a given word for a given topic, as determined during the modeling process using BERTopic.

**Fig. 3:**
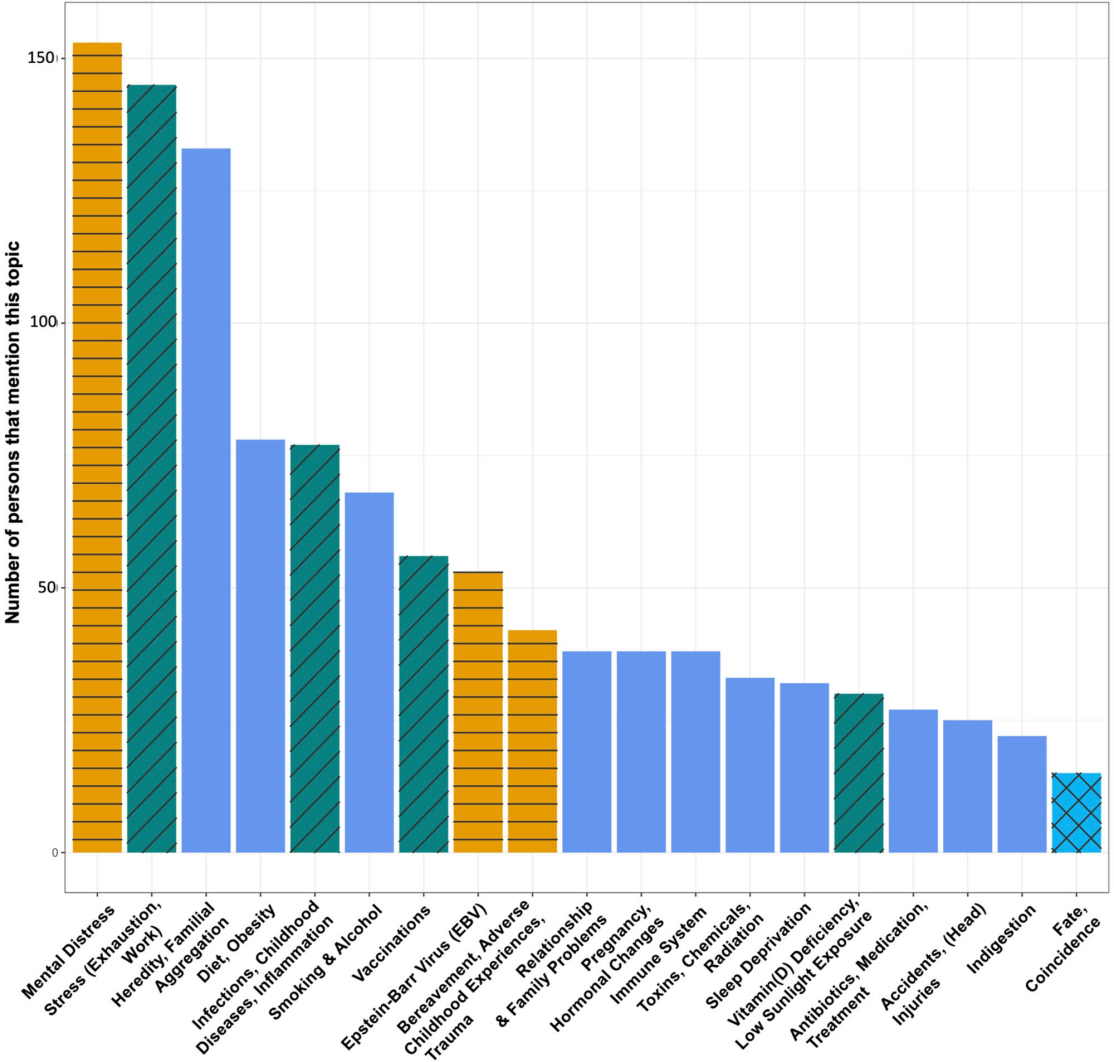
Frequency plot of theory topics. Histogram displaying the frequency of the 19 topics across all participants. The 19 topics are plotted on the x-axis, topic frequency across participants (i.e., how many participants mentioned a given topic) is plotted on the y-axis. The color scheme indicated a topic’s classification into one of four high-level categories: (1) physical health (blue; no hatching), (2) mental health (orange; horizontal lines), (3) risk factors identified in the scientific literature (turquoise; skewed lines), and (4) fate/coincidence (light blue; cross-hatched).

A word cloud of each of the 19 topics are presented in Fig. 2. The corresponding importance scores underlying the numerical format of the figure are provided in Supplementary Data 4. A detailed characterization, sample text data, and a brief overview of the current evidence base for each of the 19 topics is presented in Supplementary Data 5. The theory topics most frequently cited across all participants were Mental Distress (31.5%), Stress (Exhaustion, Work) (29.8%), Heredity/Familial Aggregation (27.4%), and Diet, Obesity’ (16.0%). Prevalence of all topics is displayed in Fig. 3, and the numerical data underlying the plot are provided in Supplementary Note 2. On average, 2.40 topics were assigned to each participant’s text data (SD = 1.60; range=1–13).

The 19 theory topics could be grouped into four high-level categories: physical health (mentioned by 56.2% of all participants), mental health (mentioned by 53.7%), risk factors established in the scientific literature (i.e., topics including genetics, EBV infection, smoking, vitamin D deficiency/low ultraviolet radiation exposure; mentioned by 47.7%), and fate/coincidence (mentioned by 3.1%). A total of 64 participants (13.2%) provided text data, some or all of which were assigned to the rare topics/unspecific mentions category. An overview of how the original topic model before merging redundant topics relates to the final topic model is provided in Supplementary Data 1.

### Topic co-occurrence

The correlation matrix, which visualizes the significant associations between topic occurrences, can be found in Supplementary Fig. 1. Notably, several important positive correlations emerged: Diet, Obesity with Smoking & Alcohol (*r* = 0.26), Stress (Exhaustion, Work) with Sleep Deprivation (*r* = 0.24), and Epstein-Barr Virus with Vitamin (D) Deficiency, Sunlight Exposure (*r* = 0.21). Overall frequencies and high-level category co-occurrence are displayed in Supplementary Fig. 2.

### Validation through thematic analysis

When we compared the topic model with the thematic analysis, we found varying degrees of overlap, which was to be expected given the different analytical methods used. Greater overlap occurred in more clearly defined content areas, such as those related to smoking, alcohol/drugs, the Epstein-Barr virus, and vaccinations. In contrast, overlap was less pronounced in more vaguely defined content areas, such as mental distress, relationship issues, trauma, and those broadly related to the immune system. Both methods of analysis showed slight variations in how these vaguely defined content areas were structured. This deviation may also be indicative of the benefits of using data-driven topic classification for exploratory purposes. A detailed description of the thematic analysis is provided in Supplementary Method 1. We also provide a mapping of thematic categories to the topic model and a manual error analysis in Supplementary Data 2.

## Discussion

This study is the first to examine the theories that persons with MS may have about the causes of their disease using a state-of-the-art topic modeling approach. Our analysis revealed 19 distinct theory topics of which Mental Distress (31.5%), Stress (Exhaustion, Work), Heredity/Familial Aggregation (27.4%), and Diet, Obesity were most prevalent. The 19 topics could be grouped into four high-level categories: physical health, mental health risk factors established in the scientific literature (i.e., topics including genetics, EBV infection, smoking, vitamin D deficiency/low ultraviolet radiation exposure), and fate/coincidence.

Our findings suggest that persons with MS associated their disease with a variety of factors. Participants often did not have elaborate theories and tended to analyze their life situation prior to the onset of MS, without necessarily assuming that these circumstances were causal. Where mentioned, theories about causes of MS were mostly based on personal experience or that of others, but for established risk factors (e.g., EBV, vitamin D) also on available official information or events, such as journal articles, information materials or events. Participants who reported having no theories or providing no information about them often did so because they believed they would not benefit from thinking about such issues, preferring instead to focus on the present. Of those who did have theories, some linked them in sophisticated ways to past life experiences, while others remained more abstract, general, and brief in their description. Participants varied in the degree to which they were convinced of their theories. Several participants also distinguished between the causes or origins of their MS, such as genetics or physical predisposition, and the triggering conditions that interacted with an underlying cause, ultimately resulting in the onset of MS. Participants also noted and reported instances of the co-occurrence or sequential occurrence of risk factors. For example, some reported that a vaccination or use of antibiotics preceded the MS symptom onset. From their perspective, there may be an intuitive inclination to attribute the symptoms to the vaccine and antibiotics.

The category of established risk factors for the MS was mentioned by 47.7% of participants. Indeed, the topic of heredity/familial aggregation was the most frequent category. The majority of participants who brought up familial risks had individuals in their family with suspected or diagnosed MS, autoimmune disorder, or other chronic diseases such as cancer of Crohn’s disease. That is, evidence-based risk factors were also reflected in the individuals’ life experiences. Of note, EBV had the fewest mentions among the four established risk factors. This is probably because our data collection largely preceded the landmark analysis by Bjornevik and colleagues^8^. Their study received much attention from the public, showing that EBV infection considerably increases the risk of subsequent MS.

What also stands out from our analysis is that around 53.7% of participants reported stress-related factors including mental distress, stress, relationship/family problems, or adverse childhood experiences as potential risk factors for MS. In some cases, traumatic events were even assumed to be causal. This shows that mental health and related experiences are highly relevant to persons with MS and are perceived as influencing their lives in many ways. This is important information as healthcare for persons with MS currently focuses heavily on treatments that are targeting physical symptoms and inflammation. Moreover, in large-scale meta-analytic studies that investigate risk factors for MS, mental health is usually not considered, despite its relevance to persons with MS (e.g.,^4,31–33^).

Consistent with the participants’ theories that mental health problems are a major risk factor for MS, previous research has found supporting evidence. For example, stress-related disorders have been associated with an increased risk of autoimmune disease^34^. In addition, mental distress and stressful events have been associated with impaired immune function^35^. While establishing causality is beyond the scope of our study, our study raises the question of how mental health relates to people’s ability to cope with MS in daily life. It may be that persons with MS have increased awareness of their mental health due to their disease burden and life history. On the other hand, it is conceivable that past distressing experiences may lead to reduced self-efficacy and a sense of heteronomous living. A coherent theory of the etiology of one’s MS may be important for gaining a sense of control over one’s life, as theorized in Cognitive Theory of Adaptation^12^. However, if one’s personal theory reinforces a self-perceived lack of control and self-efficacy, this is likely to have a negative impact on current mental and physical health^9^.

Our study also revealed several discrepancies between current scientific evidence and theories that persons with MS have about risk factors for MS. Of note was the common belief among participants that vaccinations were a risk factor for MS, despite scientific evidence to the contrary^4^. This may have direct implications for public health efforts, including vaccination campaigns. Awareness of these individual theories of MS is crucial for healthcare professionals to prevent hindered healthcare and prevention procedures, ensuring that persons with MS are not unnecessarily exposed to higher risks of vaccine-preventable diseases.

The present study has several limitations that should be considered. This study is retrospective, and participants are persons with MS, many of whom have been living with their disease for a years or decades. Looking back on their lives while being mindful of their MS and its onset is likely to influence how they remember the past and what stands out to them in retrospect. It is also likely that for some participants it may have been the first time they had thought deeply about the causes of their MS when completing the survey, whereas for others thinking about the origins of their disease may be an important topic that occurs frequently in their daily lives. This may be one reason for the observed difference in the level of detail in the participants’ theories. It is also possible that our survey may have attracted more persons with particularly strong believes that they were eager to share. However, most participants framed their theories as conclusions drawn from their life experiences and personal histories (as presented in Supplementary Data 5), making this seem unlikely. Individuals who shared their theory in the present study were more likely to be female and slightly older than registry participants who either did not have a theory or did not respond to the survey. It is a common challenge that women are often more willing to participate in studies. The slight age difference may reflect the fact that older, retired people often have more time to dedicate to research activities than their younger, employed counterparts. This is a pattern we have seen in other research we conducted with the registry as well^36^. In addition, our study focuses on people with MS’ theories about their disease, but does not examine how people’s perspectives on the etiology of MS may influence their actual health behaviors. Future research would benefit from an in-depth examination of the relationship between perspectives on etiology and individuals’ actual health practices. Finally, we note that our frequency estimates for specific theories may not necessarily be generalizable to the entire population of persons with MS in Switzerland.

Our research has both clinical and scientific implications. Our study shows that communication between healthcare professionals and persons with MS is of utmost importance. Professionals should be aware that mental health is an important issue for persons with MS in relation to the development of their disease and should ensure that adequate information about mental health and treatment options is provided to those in need. Furthermore, healthcare professionals should respect the experiences of individuals while providing them with evidence-based information about the risk factors for MS to increase their self-efficacy and support them in adopting health-promoting behaviors. Future research should pay more attention to the mental health of persons with MS – in terms of the development and course of their MS and also how current mental health and individual theories impact on self-efficacy and coping with their disease.

This study examined the theories that people with MS have about the causes of their disease using a state-of-the-art topic modeling approach. Our analysis reveals 19 distinct theory topics, with the most common being Mental Distress, Stress (Exhaustion, Work), Heredity/Familial Aggregation, and Diet, Obesity. These topics are grouped into four high-level categories: physical health, mental health, scientifically established risk factors, and fate/coincidence. Our findings suggest that people with MS associate their disease with a variety of factors, often based on personal experience or available public information, both evidence-based and non-evidence-based. Notably, 53.7% of participants reported stress-related factors as potential risk factors, indicating the high relevance of mental health for people with MS. Our research highlights the importance of communication between healthcare professionals and persons with MS—about the pathogenesis of MS, the scientific evidence for treatment, and the individual theories. Through our research, we want to draw attention to the theories of persons with MS, the importance of mental health issues for them, and encourage health professionals to engage in more patient dialog on this topic. This may have a positive effect on the individual’s adherence to treatment strategies and may also promote self-efficacy. As such, our findings are also informative in terms of support and treatment strategies for MS.

### Supplementary information

Supplementary Information

Description of Additional Supplementary Files

Supplementary Data 1

Supplementary Data 2

Supplementary Data 3

Supplementary Data 4

Supplementary Data 5

Reporting Summary

## Data Availability

We provide the survey materials in Supplementary Note 1 and anonymized excerpts of individual theories in Supplementary Data 5. To protect the privacy of our participants, the full-text data cannot be shared. The source data for Fig. 2 are provided in Supplementary Data 1. The source data underlying Fig. 3 are available in Supplementary Note 2.

## Code availability

The Python analysis script is available in Supplementary Method 2.
