# Supplementary material for: Natural language processing analysis of the theories of people with multiple sclerosis about causes of their disease
Source: Commun Med (Lond). 2024 Jun 24;4:122. doi: 10.1038/s43856-024-00546-3 (PMC11196672; doi:10.1038/s43856-024-00546-3)
Supplement: Supplementary file 1 — Supplementary Information [file 43856_2024_546_MOESM1_ESM.pdf]

Supplementary Information for:

Haag et al.: Natural language processing analysis of the theories of people with multiple sclerosis about causes of their disease

Table of Contents

Supplementary Note 1. Assessment materials 2

Supplementary Note 2. Numerical information on theory topic frequencies 4

Supplementary Method 1. Complementary information on text pre-processing and the BERTopic analysis procedure 5

Supplementary Method 2. BERTopic analysis code 9

Supplementary Figure 1. Correlation Plot of Topic Co-occurrence....12

Supplementary Figure 2: Frequency of the high-level categories and the micro-topics/unspecific mentions category 13

Supplementary Note 1. Assessment materials

Impressions and insights from persons with MS

To set the stage for the Risk Factor Questionnaire, we would like to know how you yourself see the development of MS. What do you think has contributed to or even triggered the development of MS? What risk factors do you suspect in yourself? Are there things you would avoid or change if you were younger?

Taking a citizen science approach, we want to gather as many personal ideas, impressions, and insights from persons with MS as possible. They are a very important piece of the puzzle. We know from many conversations that some memories only come into focus with time. Sometimes things that happened long before the onset of MS symptoms can suddenly become interesting.

If you would like to take this opportunity to share your impressions and insights, we would be very grateful. These questions are open-ended. You can answer them in free text.

However, if you would prefer to share your impressions and insights verbally, please feel free to call us. Either way, we look forward to hearing from you.

Have you made any assumptions about how you got MS? What are your thoughts on this?

Are there any specific risk factors that come to your mind? If so, why?'

Looking back today, were there any changes of any kind – physical, mental, or behavioral – that you observed before the onset of MS and that may have been related to the MS? This may have been a considerable time before the onset of MS. If so, how did these changes manifest and at what age did they occur?

From today's perspective, are there things you would avoid or change if you were younger?

Supplementary Note 2. Numerical information on theory topic frequencies

Mental distress: n=153; Stress (exhaustion, work): n=145; Heredity, familial aggregation: n=133; Diet: n=78; Infections, childhood diseases, inflammation: n=77; Smoking & alcohol: n=68; Vaccinations: n=56; Epstein-Barr-Virus (EBV): n=53; Bereavement, adverse childhood experiences, trauma: n=42; Relationship & family problems: n=38; Pregnancy: 38; n=Immune system: n=38; Toxins, chemicals, radiation: n=33; Sleep deprivation: n=32; Vitamin (D) deficiency, low sunlight exposure: n=30; Antibiotics, medication, treatment: n=27; Accidents, (head) injuries: n=25; Indigestion: n=22; Fate, coincidence: n=15

Supplementary Method 1. Complementary information on text pre-processing and the BERTopic analysis procedure

The following text complements the analysis description in the manuscript. For the analysis code, please refer to Supplementary Method 2.

Text preprocessing

For automatic text translation, we used the ‘French-German’ and ‘Italian-German’ open-source language models published by the Language Technology Research Group at the University of Helsinki, which can be downloaded from a website for open-source artificial intelligence (‘Hugging Face’; https://huggingface.co/Helsinki-NLP). All translations were performed locally in an offline Python environment. Corrections were made where necessary. In addition, all abbreviations in the text data were spelled out unless they were common (such as ‘MS’ for ‘multiple sclerosis’ or ‘EBV’ for ‘Epstein-Barr virus’). Participants provided the German entries in High German. Participants only very rarely used individual Swiss German words (e.g., ‘Götti’ instead of ‘Patenonkel’ [‘godfather’]) which were then transformed to High German. Occasional colloquialisms were transformed into standard language (e.g., ‘hit the bull’s eye’ to ‘is right’). We also de-identified the data by removing any names or specific locations (individuals, institutions, cities, cantons, etc.). To avoid confounding our results with unrelated information, we removed any textual data that did not relate to the question, such as individuals' current life situation (e.g., ‘I'm eating a lot of vegetables these days.’), descriptions of past situations without a direct link to their individual theories (e.g., ‘At that time, I was organizing an event that was focused on...’), or descriptions of the subsequent course of the disease (‘In the time after my diagnosis, I had several relapses and suffered from fatigue.’). As final preprocessing step, we broke the text data into text segments no longer than 128 words which was a preparation for the topic modelling procedure. The modelling procedure to be detailed in the subsequent section relies on pre-trained language models that have an upper limit on the length of sequences they can accept as input at one time, usually 128 words by default. While this limit can be extended slightly, longer strings of words are likely to contain more than one topic, and BERTopic can only assign one topic to a segment of text. Therefore, to avoid problems in the later topic modeling procedure due to mixing of topics, we broke the text data into small units of 128 words or less. Periods and commas were used to divide the text into segments in the first place. If text segments were still longer than 128 words, or if a sentence was broken into micro-segments because the commas had been used for numbering, the text segmentation was corrected manually. Finally, the text

segmentation was manually checked to ensure that sentences were broken into small units of meaning and that random breaks did not compromise the meaning of the text.

Important parameters in the BERTopic modelling procedure

In terms of the BERTopic modeling procedure, there are two important parameters that affect the final number of topics generated by BERTopic. The first parameter is the number of neighbors (*n_neighbors*) in the UMAP algorithm that we have implemented for dimensionality reduction.[1] Lower values preserve a more global structure of the data, while higher values focus more on the big picture.[2] Possible values for the number of neighbors range from two to the number of all text segments. The latter would be equivalent to assuming only a single neighborhood. In the present research, we had to strike a balance between both poles. We had a stronger emphasis on preserving the local structure of the data to keep topics smaller and more coherent, as we were interested in topic prevalence. For this reason, we sought to choose a small value for the number of neighbors. For topic modelling with BERTopic, it is recommended to iteratively explore the model with slightly different values for the number of neighbors. We iteratively explored the model for values between 5 and 25. In this way, about 1-2% of the data was considered as a neighborhood. After each model generation process, we manually checked the topics and the corresponding text data assigned to a given topic. For the final model we thus increased UMAP's default value for *n_neighbors* from 15 to 23. The reason for this choice was that, of the values tested, this value resulted in the model that best captured smaller topics, and also topics assigned to a particular category were mostly coherent.

The second important parameter, which also affects the number of topics created, is the minimum amount of text segments required for a topic to be created (*min_topic_size*).[3] Setting this value too low results in a large number of micro-topics being created, while setting it overly high risks missing less common topics. We also explored how smaller and slightly higher values changed the topic representation. For the final model, we set it to 15 text segments. This is a rather small number as 15 text segments equals around 1% of all the overall text segments sample (n=1494).

Combining similar topics for the final model

As we set the *min_topic_size* parameter to a relatively small value to ensure that no micro-topics were missed, this also resulted in a number of larger topics being decomposed into subtopics as revealed by manual exploration of the text segments. The next step was to reduce the number of themes in the model by merging those that overlapped substantially, as indicated by prior manual exploration.[4] In the Table below, we present the topics of

the original model in the right column and the topics of the final model, some of which have been merged, in the left column. Specifically, we merged the original topics 0 and 18 into 'Heredity, Familial Aggregation' (topic 1), original topics 1, 11, 15 into 'Mental Distress' (topic 5), and original topics 9, 13, 16, and 17 into 'Stress (Exhaustion, Work)' (topic 6). The final topic categories were then used for soft clustering. An overview of the mapping from the original topic model to the final reduced topic model is provided in Supplementary Data 1.

Final manual review

After the soft clustering, we performed a manual review of the classification of each text segment. The main reason for this procedure was that we were interested in topic prevalence. To do this, we first compiled a summary of the core defining content of each topic – i.e., content that was shared by the majority of text segments assigned to a given topic. The text segments were then compared with the topic summaries. If text segments clearly deviated from the core content of a topic, they were reassigned to the more appropriate category. For example, the text segment *'Mediterranean climate is better for me'* was falsely assigned to the final topic 'Toxins, chemicals, radiation' (Topic 14) and therefore manually reassigned to the Micro-topics/unspecific mentions category. Text segments that were misclassified as outliers were also reassigned to the most appropriate category. For instance, the text segment *'I was a victim of severe physical abuse*' was misclassified as Micro-topics/unspecific mentions and then manually reassigned to the category 'Bereavement, Adverse childhood experiences, Trauma'.

Validation

Given the unsupervised nature of our topic modelling analysis, we undertook a thematic analysis as a benchmark for validation. This involved coding the data, formulating and refining themes, and then defining and naming them, resulting in 31 distinct themes. These themes were then aligned with those identified in the topic modelling analysis by CH and VvW, based on overarching topic descriptions and a detailed review of the individual text data to ensure clarity of coding. This was followed by an exploration of potential factors underlying the discrepancies. We then calculated accuracy, precision, recall and F1 score.

For validation, we treat the categories identified by the thematic analysis as ground truth. Therefore, if the topic modeling analysis assigns a topic to an individual, and this assignment matches the corresponding topic assigned to the same individual in the thematic analysis, we consider the assignment made by the topic model to be 'correct'.

Accuracy: Accuracy represents the proportion of individuals correctly categorized into specific topics by the topic modelling analysis, consistent with their categorization in the thematic analysis. That is, the accuracy metric

quantifies the degree to which an individual assigned (or not assigned) to a particular topic by the topic modelling matches their assignment (or not) to the topics identified by the thematic analysis.

Precision: Precision measures the accuracy of the topic modelling analysis in identifying individuals for a given topic, as validated against the thematic analysis categories. It calculates the ratio of individuals correctly assigned to a particular theme by the topic model to the total number of individuals assigned to that theme by the Topic Model, regardless of whether the thematic analysis also identified them in the same theme. In essence, precision highlights the proportion of true positive identifications out of all positive identifications made by topic modelling.

Recall: Recall assesses the completeness of the topic modelling analysis in capturing all relevant instances for each topic, based on the thematic analysis categories. It is the proportion of individuals that both thematic analysis and topic model correctly identified for a given topic, relative to the total number of individuals identified by thematic analysis for that topic. Recall therefore indicates how many of the actual positives were successfully captured by topic modelling.

F1 Score: The F1 score provides a balance between precision and recall, providing a composite measure of the overall performance of the topic modelling analysis relative to the thematic analysis. It is the harmonic mean of Precision and Recall, providing an aggregate view of the accuracy and completeness of the topic modelling. A higher F1 score indicates a robust agreement between the topic modelling and the thematic analysis, indicating accurate and comprehensive topic assignments.

Supplementary Method 2. BERTopic analysis code

```python
#%%
# Load required modules
import pandas as pd
import numpy as np
import re
from bertopic import BERTopic
from sentence_transformers import SentenceTransformer, util
from umap import UMAP
from bertopic.vectorizers import ClassTfidfTransformer
from bertopic.representation import MaximalMarginalRelevance
from hdbscan import HDBSCAN
import spacy
from nltk.corpus import stopwords
from sklearn.feature_extraction.text import CountVectorizer
german_stop_words = stopwords.words('german') # https://github.com/stopwords-iso/stopwords-de


# Load data
df = pd.read_excel('/PATH/Theories_raw_data.xlsx', index_col=None, header=0)
theory = df['theory'].values.tolist()  # This is the column containing the text segments
id  = df['id'].values.tolist()         # This is the column containing the ids


#######################################################

### BERTOPIC Model Training

### Step 1 - Extract embeddings
# Load model suitable for German language
# https://huggingface.co/sentence-transformers/paraphrase-multilingual-MiniLM-L12-v2
model_embedding = SentenceTransformer("paraphrase-multilingual-MiniLM-L12-v2")


### Step 2 - Reduce dimensionality
umap_model = UMAP(n_neighbors=23, min_dist=0.0, metric='cosine', random_state=1)


### Step 3 - Cluster reduced embeddings
# standard HDBSCAN settings are used


### Step 4 - Tokenize topics

# Extension of the stopword list (translated into English from German)
german_stop_words.extend([# Time references
                          'year', 'week', 'day', 'time', 'period', 'month', 'day',
                          'hour', 'year', 'time',
                          # Reiteration of the question
                          'risk increase', 'risk factor', 'factor', 'risk', 'assumption',
```

```python
                              # Unspecific references to illness
                              'illness', 'illness', 'disease', 'symptom', 'problem', 'outbreak','onset',
                              # Standard expressions (in German language) to describe one's own perception/perspective
                              'background', 'role', 'denominator', 'factor', 'line', 'impression', 'thought', 'point',
                              'afterthought', 'fact', 'impression', 'perspective', 'frame',  'thing',
                              'situation', 'opinion', 'opposite', 'part'])

# Lemmatizer
nlp = spacy.load("de_core_news_sm")
# https://spacy.io/models/de
#
def lemma(text, allowed_postags=['NOUN']):  # only nouns
    text = re.sub(r'\d+', '', text)          # removal of white space
    text = re.sub(r'[,.!?+:-=`<>"/!()&%]', ' ', text) # removal of punctuation
    text = ' '.join([word.lemma_ for word in nlp(text) if word.pos_ in allowed_postags]) # lemmatization using spacy's de_core_news_sm model
    text = text.lower() # change all words to lower case
    return text

# Vectorizer
vectorizer_model= CountVectorizer(stop_words=german_stop_words, preprocessor=lemma) # remove stopwords and apply lemmatizer



### Step 5 - Create topic representation
ctfidf_model = ClassTfidfTransformer(reduce_frequent_words=True)


### Step 6 - Fine-tune topic representations using MMR
representation_model = MaximalMarginalRelevance(diversity=0.1)


### Model definition
topic_model = BERTopic(embedding_model=model_embedding,
                       representation_model=representation_model,
                       vectorizer_model=vectorizer_model,
                       umap_model=umap_model,
                       ctfidf_model=ctfidf_model,
                       n_gram_range=(1),
                       min_topic_size=15,
                       calculate_probabilities=True)

### Computing the model
topics, probabilities = topic_model.fit_transform(theory)


### Show topics
topic_model.get_topic_info()
```

```


### Following in manual review: Merging several topics into one

# Which topics shall be merged:
topics_to_merge = [[0,18],          # these topics all focus on genetics/heredity/familial aggregation
                   [1,11,15],       # these topics all focus on mental distress
                   [9,13,16,17]     # these topics all focus on work, stress
                   ]

# Merging the topics
topic_model.merge_topics(theory, topics_to_merge)

# Updating the topics and probabilities
import hdbscan
hdbscan_model = HDBSCAN(prediction_data=True)

topics= topic_model._map_predictions(topic_model.hdbscan_model.labels_)
probabilities = hdbscan.all_points_membership_vectors(topic_model.hdbscan_model)
probabilities = topic_model._map_probabilities(probabilities, original_topics=True)



########################################################
### Soft-Clustering

# Extract new topics
probability_threshold = 0.05 # Text data with probabilities below p>0.05 will not be assigned and remain outliers
# Text data is assigned to the category for which it has the highest probability of classification:
new_topics = [np.argmax(prob) if max(prob) >= probability_threshold else -1 for prob in probabilities]

# The topics allocations are then saved and corrected manually:
documents = pd.DataFrame({"id": id, "Document": theory, "topic_hard_clustering": topics, "topic_soft_clustering": new_topics})
documents.to_excel('/PATH/topic_allocation_for_manual_review.xlsx')



########################################################
### Manual review and final model

# The file with the manually corrected labels is then loaded again
documents = pd.read_excel('/PATH/topic_allocation_corrected.xlsx')
theory    = documents['Document'].values.tolist()
new_topics = documents['Topic'].values.tolist()


# Update the internal topic representation of the topics
topic_model.update_topics(theory, new_topics,  vectorizer_model=vectorizer_model)

# Update topic frequencies
documents = pd.DataFrame({"id": id, "Document": theory, "Topic": new_topics})
topic_model._update_topic_size(documents)


```

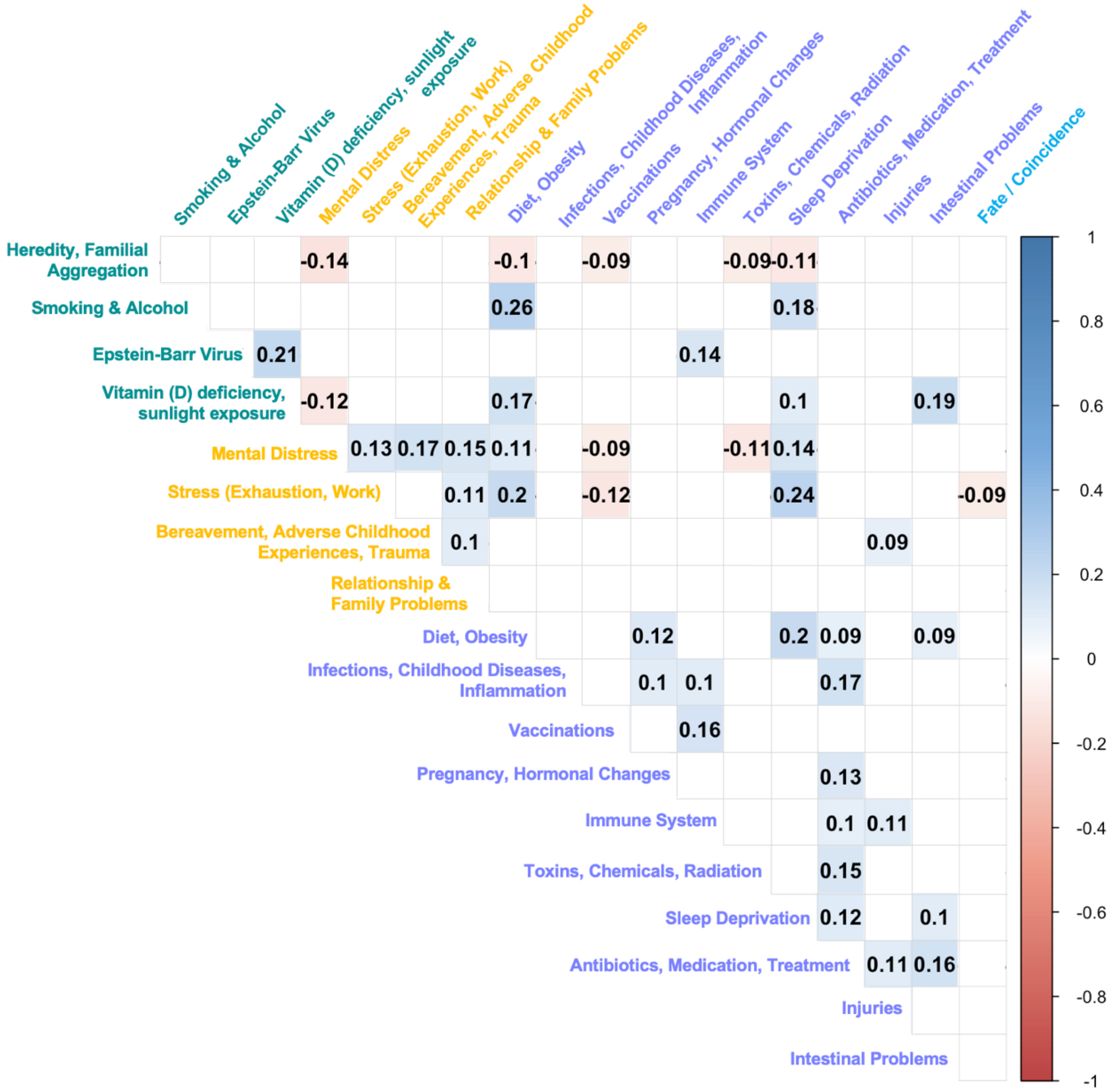

Supplementary Figure 1. Correlation Plot of Topic Co-occurrence

Correlation plot illustrating significant associations between co-occurring topics (presence vs. absence) using point-biserial correlation coefficients. Coefficients shown are statistically significant ($p<.05$); non-significant correlations are omitted. The background color indicates the direction of each correlation, with positive correlations in blue and negative correlations in red. In addition, the topic names are color-coded according to their high-level categorization: physical health (purple), mental health (orange), risk factors identified in the literature (turquoise) and fate/coincidence (light blue), and fate/coincidence (light blue).

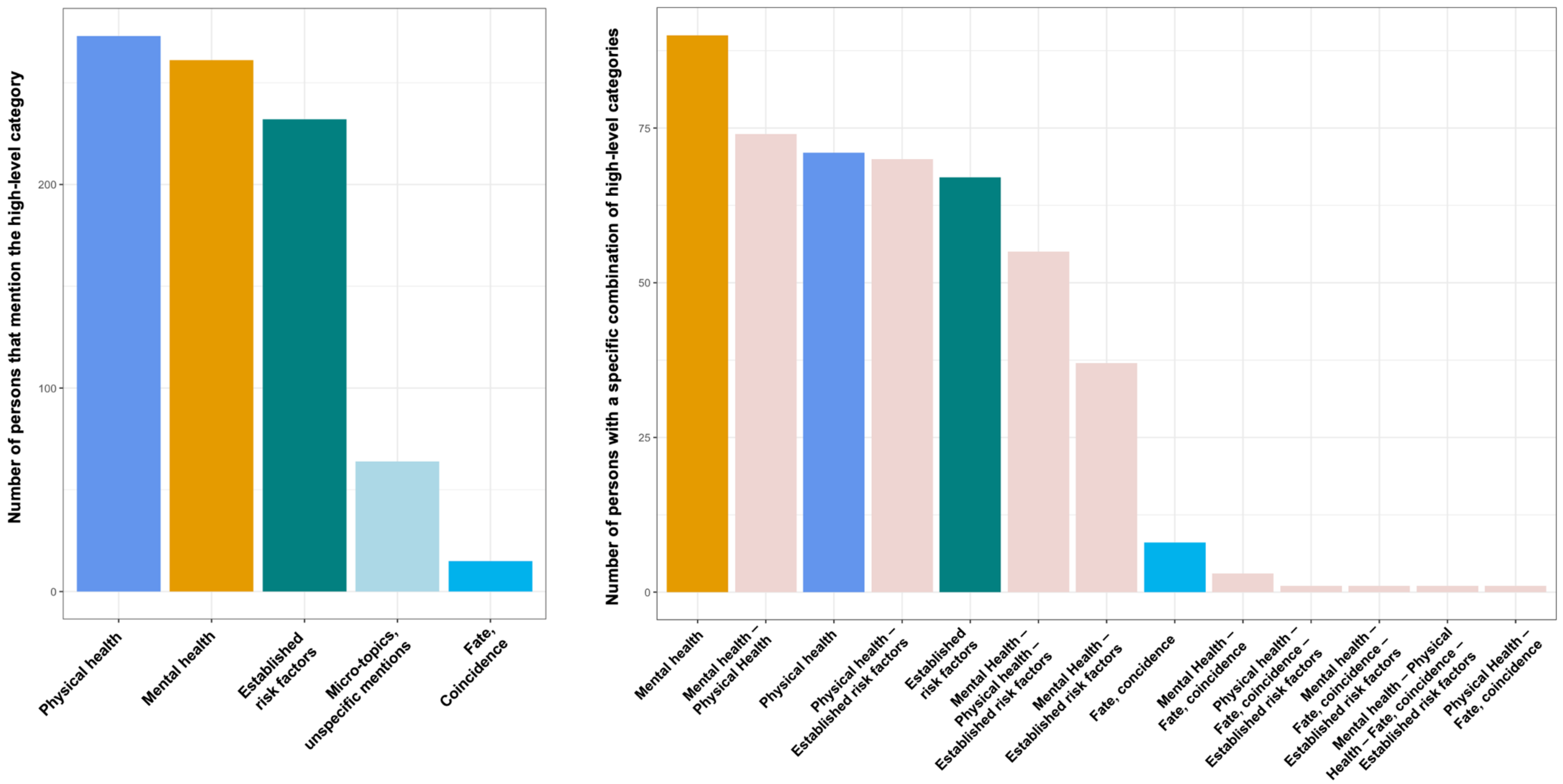

Supplementary Figure 2: Frequency of the high-level categories and the micro-topics/unspecific mentions category

Left panel: Frequency plot of the high-level categories ‘mental health’ (orange), ‘physical health’ (purple), ‘establishes risk factors’ (turquoise), ‘fate, coincidence’ (medium-blue), and the ‘micro-topics, unspecific mentions’ category (light-blue). *Right panel:* Occurrence of the four high-level categories of theory topics. Participants' responses

either contained only one category (‘mental health’ – orange, ‘physical health’ –purple, ‘establishes risk factors’ – turquoise, ‘fate, coincidence’ – medium-blue), or two or more categories co-occurred (light-pink).

References

1. Grootendorst, M. BERTopic - Dimensionality Reduction. https://maartengr.github.io/BERTopic/getting_started/dim_reduction/dim_reduction.html (2023).
2. McInnes, L., Healy, J. & Melville, J. UMAP: Uniform Manifold Approximation and Projection for Dimension Reduction. Preprint at http://arxiv.org/abs/1802.03426 (2020).
3. Grootendorst, M. BERTopic - Hyperparameter Tuning. (2023).
4. Grootendorst, M. BERTopic - Topic Reduction. (2023).
