# Supplementary material for: Natural language processing analysis of the theories of people with multiple sclerosis about causes of their disease
Source: Commun Med (Lond). 2024 Jun 24;4:122. doi: 10.1038/s43856-024-00546-3 (PMC11196672; doi:10.1038/s43856-024-00546-3)
Supplement: Supplementary file 2 — Description of Additional Supplementary Files [file 43856_2024_546_MOESM2_ESM.pdf]

Description of Additional Supplementary Files

File name: Supplementary Data 1

Description: Topics in the original and the final (reduced) topic model

File name: Supplementary Data 2

Description: Comparison of the final topic model with the thematic analysis (‘ground truth’)

File name: Supplementary Data 3

Description: Sociodemographic and clinical characteristics of participants included and excluded from the analysis

File name: Supplementary Data 4

Description: Importance scores for the unique keywords of the 19 final topics

File name: Supplementary Data 5

Description: Characterization of the 19 topics related to persons with multiple sclerosis' (MS) theories about the causes of their disease, plus a micro-topics/unspecific mentions category
