# Supplementary material for: Natural language processing analysis of the theories of people with multiple sclerosis about causes of their disease
Source: Commun Med (Lond). 2024 Jun 24;4:122. doi: 10.1038/s43856-024-00546-3 (PMC11196672; doi:10.1038/s43856-024-00546-3)
Supplement: Supplementary file 8 — Reporting Summary [file 43856_2024_546_MOESM8_ESM.pdf]

nature portfolio

Corresponding author(s): Dr. Viktor von Wyl

Last updated by author(s): Mar 20, 2024

Reporting Summary

Nature Portfolio wishes to improve the reproducibility of the work that we publish. This form provides structure for consistency and transparency in reporting. For further information on Nature Portfolio policies, see our Editorial Policies and the Editorial Policy Checklist.

Statistics

For all statistical analyses, confirm that the following items are present in the figure legend, table legend, main text, or Methods section.

n/a	Confirmed	
☐	☒	The exact sample size (*n*) for each experimental group/condition, given as a discrete number and unit of measurement
☐	☒	A statement on whether measurements were taken from distinct samples or whether the same sample was measured repeatedly
☒	☐	The statistical test(s) used AND whether they are one- or two-sided<br>*Only common tests should be described solely by name; describe more complex techniques in the Methods section.*
☒	☐	A description of all covariates tested
☒	☐	A description of any assumptions or corrections, such as tests of normality and adjustment for multiple comparisons
☐	☒	A full description of the statistical parameters including central tendency (e.g. means) or other basic estimates (e.g. regression coefficient) AND variation (e.g. standard deviation) or associated estimates of uncertainty (e.g. confidence intervals)
☒	☐	For null hypothesis testing, the test statistic (e.g. F, t, r) with confidence intervals, effect sizes, degrees of freedom and P value noted<br>*Give P values as exact values whenever suitable.*
☒	☐	For Bayesian analysis, information on the choice of priors and Markov chain Monte Carlo settings
☒	☐	For hierarchical and complex designs, identification of the appropriate level for tests and full reporting of outcomes
☒	☐	Estimates of effect sizes (e.g. Cohen's *d*, Pearson's *r*), indicating how they were calculated

Our web collection on statistics for biologists contains articles on many of the points above.

Software and code

Policy information about availability of computer code

Data collection	The data used for this study were collected by the Swiss Multiple Sclerosis Registry, a longitudinal research study. Pseudonymized data were collected using an in-house developed, noncommercial survey tool.
Data analysis	The Python analysis script is available in Supplementary Method 2.

For manuscripts utilizing custom algorithms or software that are central to the research but not yet described in published literature, software must be made available to editors and reviewers. We strongly encourage code deposition in a community repository (e.g. GitHub). See the Nature Portfolio guidelines for submitting code & software for further information.

Data

Policy information about availability of data

All manuscripts must include a data availability statement. This statement should provide the following information, where applicable:

- Accession codes, unique identifiers, or web links for publicly available datasets
- A description of any restrictions on data availability
- For clinical datasets or third party data, please ensure that the statement adheres to our policy

We provide the survey materials in Supplementary Note 1 and anonymized excerpts of individual theories in Supplementary Data 5. To protect the privacy of our participants, the full-text data cannot be shared. The source data for Figure 2 are provided in Supplementary Data 1. The source data underlying Figure 3 are available in Supplementary Note 2.

Human research participants

Policy information about studies involving human research participants and Sex and Gender in Research.

Reporting on sex and gender	We report on participants' sex (self-reported at baseline). Our study examines individual theories held by people with multiple sclerosis about the causes of their disease. Most of our participants were female, which parallels multiple sclerosis being more common in women. However, we did not perform stratified analyses by sex/gender as this was not within the scope of our primary research objective.
Population characteristics	We have reported relevant characteristics of the population with respect to age, sex, and disease characteristics. All participants of the Swiss Multiple Sclerosis Registry reside in Switzerland.
Recruitment	Participants were enrolled in the Swiss Multiple Sclerosis Registry. Data were collected as part of the registry's biannual assessments, so no separate recruitment was necessary.
Ethics oversight	The Swiss Multiple Sclerosis Registry has been approved by th Ethics Committee of the Canton of Zurich (PB-2016- 00894, BASEC2019-01027)

Note that full information on the approval of the study protocol must also be provided in the manuscript.

Field-specific reporting

Please select the one below that is the best fit for your research. If you are not sure, read the appropriate sections before making your selection.

☐ Life sciences ☒ Behavioural & social sciences ☐ Ecological, evolutionary & environmental sciences

For a reference copy of the document with all sections, see nature.com/documents/nr-reporting-summary-flat.pdf

Behavioural & social sciences study design

All studies must disclose on these points even when the disclosure is negative.

Study description	This is a cross-sectional study that analyzes qualitative data (text) using a quantitative approach (topic modeling).
Research sample	Participants were recruited from the Swiss Multiple Sclerosis (MS) Registry, which includes persons aged 18 years and older with MS who are living or receiving care in Switzerland. Data were collected as part of regular follow-up. The Swiss MS Registry is considered to be approximately representative of the Swiss population of people with MS, with exceptions described and discussed in detail elsewhere, https://doi.org/10.3389/fneur.2019.00953.
Sampling strategy	All participants of the Swiss Multiple Sclerosis (MS) Registry (n = ~2700, status March 2023) were invited to participate in the present study. A total of 603 individuals participated in the survey about theories of the origin of their MS. Of these, 107 participants either provided no response or indicated in their free-text response that they did not have a hypothesis about the cause of their illness and were therefore not included in this analysis. Participants who reported having no theories often indicated that they would not benefit from thinking about such issues, preferring instead to focus on the present. The responses of a further 10 participants could not be classified, either because they indicated that they had a theory but provided no further details, or because their responses did not directly address the questions (but included, for example, symptom descriptions of the early stages of their MS). This resulted in a final sample of 486 participants whose text responses form the basis of this study. The mean age of the final study sample participants was 52.15 years (standard deviation, SD=12.48 years; range: 21-86) and 80.3% were female.<br><br>Sample size was not predefined, but determined by the number of registry participants who completed the survey. Based on the registry's extensive experience with its previous surveys, we expected the response rate to be roughly in this range. In terms of the appropriateness of the data analysis approach, we conducted a preliminary manual review of the final sample of textual data collected, ensuring that a rich variety of individual insights and experiences were captured. From a data analysis point of view, topic modelling as implemented in BERTopic can handle different amounts of textual data, which makes BERTopic a suitable tool for our analysis. While there is still a possibility that very rare topics may not appear in the final topic model, but instead in a summary category of rare topics, we also present detailed information about this category to counteract any such bias.
Data collection	The survey was part of the SMSR's 'Risk Factors Project' and assessed participants' individual theories about the causes of their MS based on open-ended questions (the full survey is available in Supplementary Material S1). The present study is based on the following two survey questions, that assessed which general (question 1) and specific (question 2) risk factors participants believed had caused their MS: (1) 'Have you made any assumptions about how you developed MS? What do you think?' and question (2) Specific risk factors 'Are there any specific risk factors that come to your mind? If so, why?'. Question 1 was designed as an introduction to familiarize participants with the topic of theories about the cause of their MS, while question 2 was designed as a follow-up question to elicit more detail about the specific nature of the risk factors and possible underlying mechanisms. However, many participants provided a comprehensive answer to question 1 that also covered question 2. In this case, participants either left question 2 blank or briefly repeated what they had written before. Given these response patterns, the text data from both questions were combined for subsequent analysis.

	While most registry participants complete surveys online using the registry's proprietary software, some still prefer to complete paper surveys that are later digitized by the registry.
Timing	The present research is based on a survey assessing participants' individual theories about the causes of their MS. The survey was part of the SMSR's 'Risk Factors Project' which was launched in 2020 and data collection continued until March 2023.
Data exclusions	A total of 603 individuals participated in the survey about theories of the origin of their MS. Of these, 107 participants either provided no response or indicated in their free-text response that they did not have a hypothesis about the cause of their illness and were therefore not included in this analysis. For more information, please see section 'Sampling strategy.'
Non-participation	As this was a cross-sectional study, there were no dropouts. Of all the registry participants contacted (n = ~2700, as of March 2023), only 603 responded to the survey. This response rate is consistent with trends observed in previous registry projects. For more information, please see section 'Sampling strategy'
Randomization	This is an observational study.

Reporting for specific materials, systems and methods

We require information from authors about some types of materials, experimental systems and methods used in many studies. Here, indicate whether each material, system or method listed is relevant to your study. If you are not sure if a list item applies to your research, read the appropriate section before selecting a response.

Materials & experimental systems

n/a	Involved in the study
☒	☐ Antibodies
☒	☐ Eukaryotic cell lines
☒	☐ Palaeontology and archaeology
☒	☐ Animals and other organisms
☒	☐ Clinical data
☒	☐ Dual use research of concern

Methods

n/a	Involved in the study
☒	☐ ChIP-seq
☒	☐ Flow cytometry
☒	☐ MRI-based neuroimaging
